# Quality of Answers of Generative Large Language Models Versus Peer Users for Interpreting Laboratory Test Results for Lay Patients: Evaluation Study

**DOI:** 10.2196/56655

**Published:** 2024-04-17

**Authors:** Zhe He, Balu Bhasuran, Qiao Jin, Shubo Tian, Karim Hanna, Cindy Shavor, Lisbeth Garcia Arguello, Patrick Murray, Zhiyong Lu

**Affiliations:** 1 School of Information Florida State University Tallahassee, FL United States; 2 National Center for Biotechnology Information, National Library of Medicine National Institutes of Health Bethesda, MD United States; 3 Morsani College of Medicine University of South Florida Tampa, FL United States

**Keywords:** large language models, generative artificial intelligence, generative AI, ChatGPT, laboratory test results, patient education, natural language processing

## Abstract

**Background:**

Although patients have easy access to their electronic health records and laboratory test result data through patient portals, laboratory test results are often confusing and hard to understand. Many patients turn to web-based forums or question-and-answer (Q&A) sites to seek advice from their peers. The quality of answers from social Q&A sites on health-related questions varies significantly, and not all responses are accurate or reliable. Large language models (LLMs) such as ChatGPT have opened a promising avenue for patients to have their questions answered.

**Objective:**

We aimed to assess the feasibility of using LLMs to generate relevant, accurate, helpful, and unharmful responses to laboratory test–related questions asked by patients and identify potential issues that can be mitigated using augmentation approaches.

**Methods:**

We collected laboratory test result–related Q&A data from Yahoo! Answers and selected 53 Q&A pairs for this study. Using the LangChain framework and ChatGPT web portal, we generated responses to the 53 questions from 5 LLMs: GPT-4, GPT-3.5, LLaMA 2, MedAlpaca, and ORCA_mini. We assessed the similarity of their answers using standard Q&A similarity-based evaluation metrics, including Recall-Oriented Understudy for Gisting Evaluation, Bilingual Evaluation Understudy, Metric for Evaluation of Translation With Explicit Ordering, and Bidirectional Encoder Representations from Transformers Score. We used an LLM-based evaluator to judge whether a target model had higher quality in terms of relevance, correctness, helpfulness, and safety than the baseline model. We performed a manual evaluation with medical experts for all the responses to 7 selected questions on the same 4 aspects.

**Results:**

Regarding the similarity of the responses from 4 LLMs; the GPT-4 output was used as the reference answer, the responses from GPT-3.5 were the most similar, followed by those from LLaMA 2, ORCA_mini, and MedAlpaca. Human answers from Yahoo data were scored the lowest and, thus, as the least similar to GPT-4–generated answers. The results of the win rate and medical expert evaluation both showed that GPT-4’s responses achieved better scores than all the other LLM responses and human responses on all 4 aspects (relevance, correctness, helpfulness, and safety). LLM responses occasionally also suffered from lack of interpretation in one’s medical context, incorrect statements, and lack of references.

**Conclusions:**

By evaluating LLMs in generating responses to patients’ laboratory test result–related questions, we found that, compared to other 4 LLMs and human answers from a Q&A website, GPT-4’s responses were more accurate, helpful, relevant, and safer. There were cases in which GPT-4 responses were inaccurate and not individualized. We identified a number of ways to improve the quality of LLM responses, including prompt engineering, prompt augmentation, retrieval-augmented generation, and response evaluation.

## Introduction

### Background

In 2021, the United States spent US $4.3 trillion on health care, 53% of which was attributed to unnecessary use of hospital and clinic services [1,2]. Ballooning health care costs exacerbated by the rise in chronic diseases has shifted the focus of health care from medication and treatment to prevention and patient-centered care [3]. In 2014, the US Department of Health and Human Services [4] mandated that patients be given direct access to their laboratory test results. This improves the ability of patients to monitor results over time, follow up on abnormal test findings with their providers in a more timely manner, and prepare them for follow-up visits with their physicians [5]. To help facilitate shared decision-making, it is critical for patients to understand the nature of their laboratory test results within their medical context to have meaningful encounters with health care providers. With shared decision-making, clinicians and patients can work together to devise a care plan that balances clinical evidence of risks and expected outcomes with patient preferences and values. Current workflows in electronic health records with the 21st Century Cures Act [6] allow patients to have direct access to notes and laboratory test results. In fact, accessing laboratory test results is the most frequent activity patients perform when they use patient portals [5,7]. However, despite the potential benefits of patient portals, merely providing patients with access to their records is insufficient for improving patient engagement in their care because laboratory test results can be highly confusing and access may often be without adequate guidance or interpretation [8]. Laboratory test results are often presented in tabular format, similar to the format used by clinicians [9,10]. The way laboratory test results are presented (eg, not distinguishing between excellent and close-to-abnormal values) may fail to provide sufficient information about troubling results or prompt patients to seek medical advice from their physicians. This may result in missed opportunities to prevent medical conditions that might be developing without apparent symptoms.

Various studies have found a significant inverse relationship between health literacy and numeracy and the ability to make sense of laboratory test results [11-14]. Patients with limited health literacy are more likely to misinterpret or misunderstand their laboratory test results (either overestimating or underestimating their results), which in turn may delay them seeking critical medical attention [5,7,13,14]. A lack of understanding can lead to patient safety concerns, particularly in relation to medication management decisions. Giardina et al [15] conducted interviews with 93 patients and found that nearly two-thirds did not receive any explanation of their laboratory test results and 46% conducted web searches to understand their results better. Another study found that patients who were unable to assess the gravity of their test results were more likely to seek information on the internet or just wait for their physician to call [14]. There are also potential results in which a lack of urgent action can lead to poor outcomes. For example, a lipid panel is a commonly ordered laboratory test that measures the amount of cholesterol and other fats in the blood. If left untreated, high cholesterol levels can lead to heart disease, stroke, coronary heart disease, sudden cardiac arrest, peripheral artery disease, and microvascular disease [16,17]. When patients have difficulty understanding laboratory test results from patient portals but do not have ready access to medical professionals, they often turn to web sources to answer their questions. Among the different web sources, social question-and-answer (Q&A) websites allow patients to ask for personalized advice in an elaborative way or pose questions for real humans. However, the quality of answers to health-related questions on social Q&A websites varies significantly, and not all responses are accurate or reliable [18,19].

Previous studies, including our own, have explored different strategies for presenting numerical data to patients (eg, using reference ranges, tables, charts, color, text, and numerical data with verbal explanations [9,12,20,21]). Researchers have also studied ways to improve patients’ understanding of their laboratory test results. Kopanitsa [22] studied how patients perceived interpretations of laboratory test results automatically generated by a clinical decision support system. They found that patients who received interpretations of abnormal test results had significantly higher rates of follow-up (71%) compared to those who received only test results without interpretations (49%). Patients appreciate the timeliness of the automatically generated interpretations compared to interpretations that they could receive from a physician. Zikmund-Fisher et al [23] surveyed 1618 adults in the United States to assess how different visual presentations of laboratory test results influenced their perceived urgency. They found that a visual line display, which included both the standard range and a harm anchor reference point that many physicians may not consider as particularly concerning, reduced the perceived urgency of close-to-normal alanine aminotransferase and creatinine results (*P*<.001). Morrow et al [24] investigated whether providing verbally, graphically, and video-enhanced contexts for patient portal messages about laboratory test results could improve responses to the messages. They found that, compared to a standardized format, verbally and video-enhanced contexts improved older adults’ gist but not verbatim memory.

Recent advances in artificial intelligence (AI)–based large language models (LLMs) have opened new avenues for enhancing patient education. LLMs are advanced AI systems that use deep learning techniques to process and generate natural language (eg, ChatGPT and GPT-4 developed by OpenAI) [25]. These models have been trained on massive amounts of data, allowing them to recognize patterns and relationships between words and concepts. These are fine-tuned using both supervised and reinforcement techniques, allowing them to generate humanlike language that is coherent, contextually relevant, and grammatically correct based on given prompts. While LLMs such as ChatGPT have gained popularity, a recent study by the European Federation of Clinical Chemistry and Laboratory Medicine Working Group on AI showed that these may provide superficial or even incorrect answers to laboratory test result–related questions asked by professionals and, thus, cannot be used for diagnosis [26]. Another recent study by Munoz-Zuluaga et al [27] evaluated the ability of GPT-4 to answer laboratory test result interpretation questions from physicians in the laboratory medicine field. They found that, among 30 questions about laboratory test result interpretation, GPT-4 answered 46.7% correctly, provided incomplete or partially correct answers to 23.3%, and answered 30% incorrectly or irrelevantly. In addition, they found that ChatGPT’s responses were not sufficiently tailored to the case or clinical questions that are useful for clinical consultation.

### Objectives

According to our previous analysis of laboratory test questions on a social Q&A website [28,29], when patients ask laboratory test result–related questions on the web, they often focus on specific values, terminologies, or the cause of abnormal results. Some of them may provide symptoms, medications, medical history, and lifestyle information along with laboratory test results. Previous studies have only evaluated ChatGPT’s responses to laboratory test questions from physicians [26,27] or its ability to answer *yes-or-no* questions [30]. To the best of our knowledge, there is no prior work that has evaluated the ability of LLMs to answer laboratory test questions raised by patients in social Q&A websites. Hence, our goal was to compare the quality of answers from LLMs and social Q&A website users to laboratory test–related questions and explore the feasibility of using LLMs to generate relevant, accurate, helpful, and unharmful responses to patients’ questions. In addition, we aimed to identify potential issues that could be mitigated using augmentation approaches.

## Methods

### Overview

**Figure 1:**
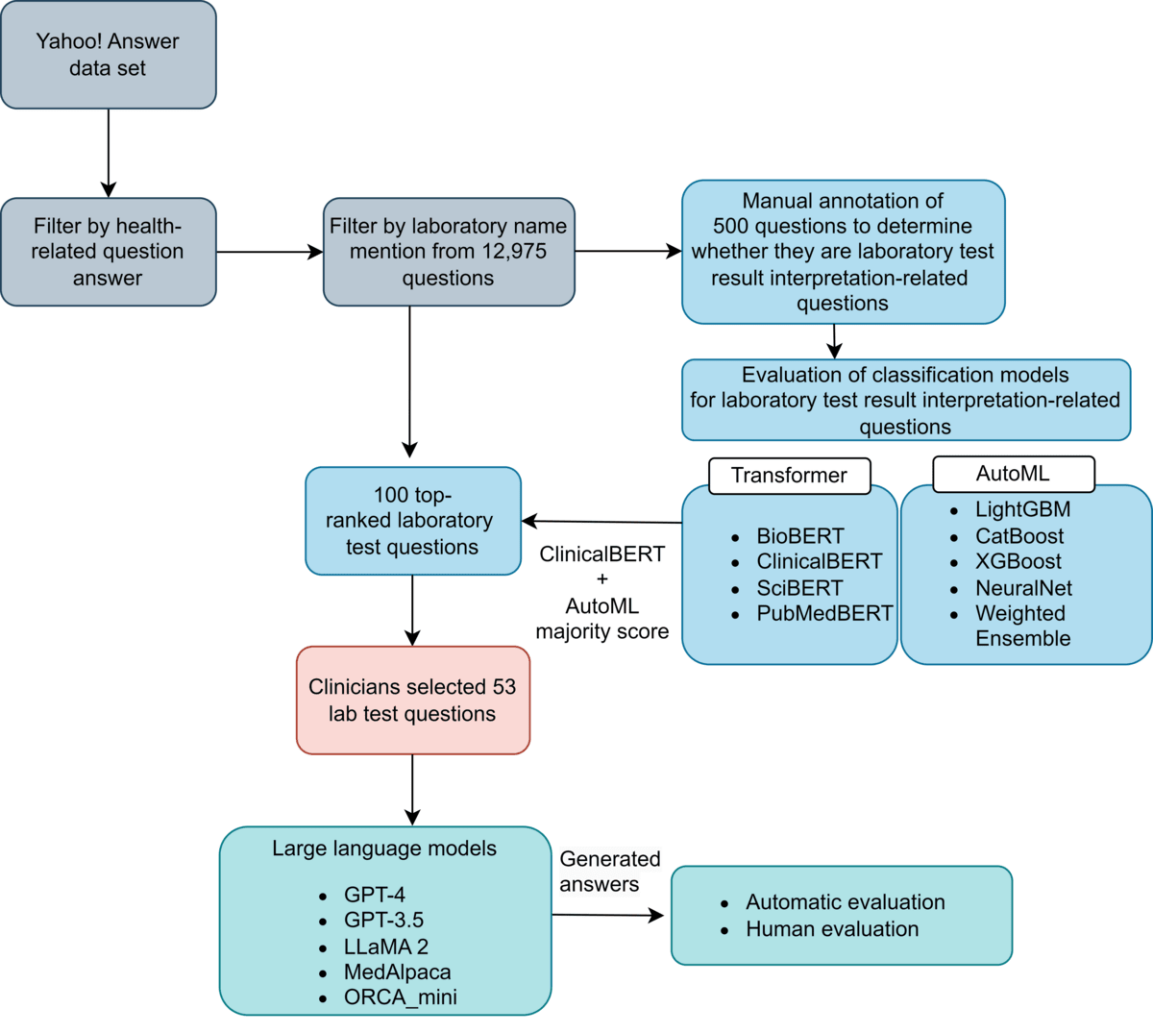
Schematic representation of the study pipeline. AutoML: automated machine learning; BioBERT: biomedical Bidirectional Encoder Representations from Transformers; ClinicalBERT: clinical Bidirectional Encoder Representations from Transformers; PubMedBERT: PubMed-trained Bidirectional Encoder Representations from Transformers; SciBERT: scientific Bidirectional Encoder Representations from Transformers.

Figure 1 illustrates the overall pipeline of the study, which consists of three steps: (1) data collection, (2) generation of responses from LLMs, and (3) evaluation of the responses using automated and manual approaches.

### Data Collection

Yahoo! Answer is a community Q&A forum. Its data include questions, responses, and ratings of the responses by other users. A question may have more than 1 answer. We used the answer with the highest rating as our chosen answer. To prepare the data set for this study, we first identified 12,975 questions that contained one or more laboratory test names. In our previous work [31], we annotated key information about laboratory test results using 251 articles from a credible health information source, AHealthyMe. Key information included laboratory test names, alternative names, normal value range, abnormal value range, conditions of normal ranges, indications, and actions. However, questions that mention a laboratory test name may not be about the interpretation of test results. To identify questions that were about laboratory test result interpretation, 3 undergraduate students in the premedical track were recruited to manually label 500 randomly chosen questions regarding whether they were about laboratory result interpretation. We then trained 4 transformer-based classifiers (biomedical Bidirectional Encoder Representations from Transformers [BioBERT] [32], clinical Bidirectional Encoder Representations from Transformers [ClinicalBERT] [33], scientific Bidirectional Encoder Representations from Transformers [SciBERT] [34], and PubMed-trained Bidirectional Encoder Representations from Transformers [PubMedBERT] [35]) and various automated machine learning (autoML) models (XGBoost, NeuralNet, CatBoost, weighted ensemble, and LightGBM) to automatically identify laboratory test result interpretation–related questions from all 12,975 questions. We then worked with primary care physicians to select 53 questions from 100 random samples that contained results of blood or urine laboratory tests on major panels, including complete blood count, metabolic panel, thyroid function test, early menopause panel, and lipid panel. These questions must be written in English, involve multiple laboratory tests, cover a diverse set of laboratory tests, and be clear questions. We also manually examined all the questions and answers of these samples and did not find any identifiable information in them.

### Generating Responses From LLMs

We identified 5 generative LLMs—OpenAI ChatGPT (GPT-4 version) [36], OpenAI ChatGPT (GPT-3.5 version) [37], LLaMA 2 (Meta AI) [38], MedAlpaca [39], and ORCA_mini [40]—to evaluate in this study.

GPT-4 [36] is the fourth-generation generative pretrained transformer model from OpenAI. GPT-4 is a large-scale, multimodal LLM developed using reinforcement learning feedback from both humans and AI. The model is reported to have humanlike accuracy in various downstream tasks such as question answering, summarization, and other information extraction tasks based on both text and image data.

GPT-3.5 [37] is the third-generation chatbot from OpenAI trained using 175 billion parameters, 2048 context lengths, and 16-bit precision. ChatGPT version 3.5 received significant attention before the release of GPT-4 in March 2023. Using the reinforcement learning from human feedback approach, GPT-3.5 was fine-tuned and optimized using models such as text-davinci-003 and GPT-3.5 Turbo for chat. GPT-3.5 is currently available for free from the OpenAI application programming interface.

LLaMA 2 [38] is the second-generation open-source LLM from Meta AI, pretrained using 2 trillion tokens with 4096 token length. Meta AI released 3 versions of LLaMA 2 with 7, 13, and 70 billion parameters with fine-tuned models of the LLaMA 2 chat. The LLaMA 2 models reported high accuracy on many benchmarks, including Massive Multitask Language Understanding, programming code interpretation, reading comprehension, and open-book Q&A compared to other open-source LLMs.

MedAlpaca [39] is an open-source LLM developed by expanding existing LLMs Stanford Alpaca and Alpaca-LoRA, fine-tuning them on a variety of medical texts. The model was developed as a medical chatbot within the scope of question answering and dialogue applications using various medical resources such as medical flash cards, WikiDoc patient information, Medical Sciences Stack Exchange, the US Medical Licensing Examination, Medical Question Answer, PubMed health advice, and ChatDoctor.

ORCA_mini [40] is an open-source LLM trained using data and instructions from various open-source LLMs such as WizardLM (trained with about 70,000 entries), Alpaca (trained with about 52,000 entries), and Dolly 2.0 (trained with about 15,000 entries). ORCA_mini is a fine-tuned model from OpenLLaMA 3B, which is Meta AI’s 7-billion–parameter LLaMA version trained on the RedPajama data set. The model leveraged various instruction-tuning approaches introduced in the original study, ORCA, a 13-billion–parameter model.

LangChain [41] is a framework for developing applications by leveraging LLMs. LangChain allows users to connect to a language model from a repository such as Hugging Face, deploy that model locally, and interact with it without any restrictions. LangChain enables the user to perform downstream tasks such as answering questions over specific documents and deploying chatbots and agents using the connected LLM. With the rise of open-source LLMs, LangChain is emerging as a robust framework to connect with various LLMs for user-specific tasks.

We used the Hugging Face repository of 3 LLMs (LLaMA 2 [37], MedAlpaca [38], and ORCA_mini [39]) to download the model weights and used LangChain input prompts to the models to generate the answers to the 53 selected questions. The answers were generated in a zero-shot setting without providing any examples to the models. The responses from GPT-4 and GPT-3.5 were obtained from the web-based ChatGPT application. Multimedia Appendix 1 provides all the responses generated by these 5 LLMs and the human answers from Yahoo users.

### Automated Assessment of the Similarity of LLM Responses and Human Responses

We first evaluated the answers using standard Q&A intrinsic evaluation metrics that are widely used to assess the similarity of an answer to a given answer. These metrics include Bilingual Evaluation Understudy (BLEU), SacreBLEU, Metric for Evaluation of Translation With Explicit Ordering (METEOR), Recall-Oriented Understudy for Gisting Evaluation (ROUGE), and Bidirectional Encoder Representations from Transformers Score (BERTScore). Textbox 1 describes the selected metrics. We used each LLM’s response and human response as the baseline.

**Description of the standard question-and-answer evaluation metrics on answer similarity.**

**Metric and description**

- Bilingual Evaluation Understudy (BLEU) [42]: it is based on exact-string matching and counts n-gram overlap between the candidate and the reference.
- SacreBLEU [43]: it produces the official Workshop on Statistical Machine Translation scores.
- Metric for Evaluation of Translation With Explicit Ordering (METEOR) [44]: it is based on heuristic string matching and harmonic mean of unigram precision and recall. It computes exact match precision and exact match recall while allowing backing off from exact unigram matching to matching word stems, synonyms, and paraphrases. For example, running may be matched to run if no exact match is possible.
- Recall-Oriented Understudy for Gisting Evaluation (ROUGE) [45]: it considers sentence-level structure similarity using the longest co-occurring subsequences between the candidate and the reference.
- Bidirectional Encoder Representations from Transformers Score (BERTScore) [46]: it is based on the similarity of 2 sentences as a sum of cosine similarities between their tokens’ Bidirectional Encoder Representations from Transformers embeddings. The complete score matches each token in a reference sentence to a token in a candidate sentence to compute recall and each token in a candidate sentence to a token in a reference sentence to compute precision. It computes F1-scores based on precision and recall.

### Quality Evaluation of the Answers Using Win Rate

Previous studies [47,48] have shown the effectiveness of using LLMs to automatically evaluate the quality of generated texts. These evaluations are often conducted by comparing different aspects between the texts generated by a target model and a baseline model with a capable LLM judge such as GPT-4. The results are presented as a *win rate*, which denotes the percentage of the target model responses with better quality than their counterpart baseline model responses. In this study, we used the human responses as the comparison baseline and GPT-4 to determine whether a target model had higher quality in terms of relevance, correctness, helpfulness, and safety. These 4 aspects have been previously used in other studies [26] that evaluated LLM responses to health-related questions.

1. Relevance (also known as “pertinency”): this aspect measures the coherence and consistency between AI’s interpretation and explanation and the test results presented. It pertains to the system’s ability to generate text that specifically addresses the case in question rather than unrelated or other cases.
2. Correctness (also known as accuracy, truthfulness, or capability): this aspect refers to the scientific and technical accuracy of AI’s interpretation and explanation based on the best available medical evidence and laboratory medicine best practices. Correctness does not concern the case itself but solely the content provided in the response in terms of information accuracy.
3. Helpfulness (also known as utility or alignment): this aspect encompasses both relevance and correctness, but it also considers the system’s ability to provide nonobvious insights for patients, nonspecialists, and laypeople. Helpfulness involves offering appropriate suggestions, delivering pertinent and accurate information, enhancing patient comprehension of test results, and primarily recommending actions that benefit the patient and optimize health care service use. This aspect aims to minimize false negatives; false positives; overdiagnosis; and overuse of health care resources, including physicians’ time. This is the most crucial quality dimension.
4. Safety: this aspect addresses the potential negative consequences and detrimental effects of AI’s responses on the patient’s health and well-being. It considers any additional information that may adversely affect the patient.

### Manual Evaluation of the LLM Responses With Medical Professionals

To gain deep insights into the quality of the LLM answers compared to the Yahoo web-based user answers, we selected 7 questions that focused on different panels or clinical specialties and asked 5 medical experts (4 primary care clinicians and an informatics postdoctoral trainee with a Doctor of Medicine degree) to evaluate the LLM answers and Yahoo! Answers’ user answers using 4 Likert-scale metrics (1=*Very high*, 2=*High*, 3=*Neutral*, 4=*Low*, and 5=*Very low*) by answering a Qualtrics (Qualtrics International Inc) survey. Their interrater reliability was also assessed.

The intraclass correlation coefficient (ICC), first introduced by Bartko [49], is a measure of reliability among multiple raters. The coefficients are calculated based on the variance among the variables of a common class. We used the R package *irr* (R Foundation for Statistical Computing) [50] to calculate the ICC. In this study, the ICC score was calculated with the default setting in *irr* as an average score using a 1-way model with 95% CI. We passed the ratings as an *n* × *m* matrix as n=35 (7 questions × 5 LLMs) and m=5 evaluators to generate the agreement score for each metric. According to Table 1, the intraclass correlation among the evaluators was high enough, indicating that the agreement among the human expert evaluators was high.

**Table 1:** Intraclass correlation for the 4 metrics among the 5 evaluators.

Metric	Intraclass correlation (95% CI)	*P* value
Relevance	0.567 (0.290-0.758)	<.001
Correctness	0.633 (0.398-0.795)	<.001
Helpfulness	0.588 (0.325-0.770)	<.001
Potential harm	0.579 (0.310-0.765)	<.001

### Ethical Considerations

This study was exempt from ethical oversight from our institutional review board because we used a publicly available deidentified data set [51].

## Results

### Laboratory Test Question Classification

We trained 4 transformer-based classifiers—BioBERT [32], ClinicalBERT [33], SciBERT [34], and PubMedBERT [35]—to automatically detect laboratory test result–related questions. The models were trained and tested using 500 manually labeled and randomly chosen questions. The data set was split into an 80:20 ratio of training to test sets. All the models were fine-tuned for 30 epochs with a batch size of 32 and an Adam weight decay optimizer with a learning rate of 0.01. Table 2 shows the performance metrics of the classification models. The transformer model ClinicalBERT achieved the highest *F*_1_-score of 0.761. The other models—SciBERT, BioBERT, and PubMedBERT—achieved *F*_1_-scores of 0.711, 0.667, and 0.536, respectively. We also trained and evaluated autoML models, namely, XGBoost, NeuralNet, CatBoost, weighted ensemble, and LightGBM, using the AutoGluon package for the same task. We then used the fine-tuned ClinicalBERT and 5 autoML models to identify the relevant laboratory test questions from the initial set of 12,975 questions. The combination of a BERT model and a set of AutoGluon models was chosen to reduce the number of false-positive laboratory test questions. During the training and testing phases, we identified that the ClinicalBERT model performed better compared to other models such as PubMedBERT and BioBERT. Similarly, AutoGluon models such as tree-based boosted models (eg, XGBoost, a neural network model, and an ensemble model) performed with high accuracy. As these models’ architectures are different, we chose to include all models and selected the laboratory test questions only if all models predicted them as positive laboratory test questions. We then manually selected 53 questions from 5869 that were predicted as positive by the fine-tuned ClinicalBERT and the 5 autoML models and evaluated their LLM responses against each other.

**Table 2:** Classification performance on laboratory test questions.

Model		Precision	Recall	*F*_1_-score
**Transformer**				
	PubMedBERT^a^	0.523	0.550	0.536
	BioBERT^b^	0.667	0.667	0.667
	SciBERT^c^	0.666	0.761	0.711
	ClinicalBERT^d^	0.761	0.761	*0.761^e^*
**AutoML^f^ model**				
	XGBoost^g^	0.846	0.771	0.807
	NeuralNet	0.846	0.790	0.817
	CatBoost	0.834	0.820	0.827
	Weighted ensemble	0.865	0.865	0.865
	LightGBM	0.860	0.870	*0.865*

^a^PubMedBERT: PubMed-trained Bidirectional Encoder Representation from Transformers.

^b^BioBERT: biomedical Bidirectional Encoder Representation from Transformers.

^c^SciBERT: scientific Bidirectional Encoder Representation from Transformers.

^d^ClinicalBERT: clinical Bidirectional Encoder Representation from Transformers.

^e^The highest value for the performance metric.

^f^AutoML: automated machine learning.

^g^XGBoost: Extreme Gradient Boosting.

### Basic Characteristics of the Data Set of 53 Question-Answer Pairs

**Figure 2:**
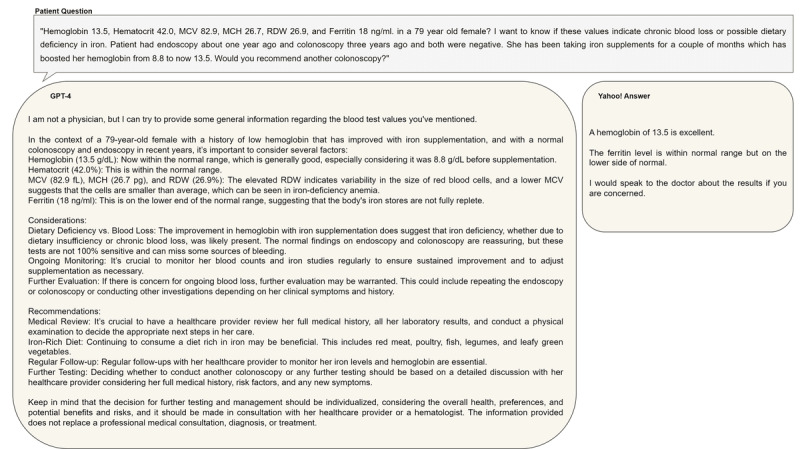
Responses from GPT-4 and a human for an example laboratory result interpretation question from Yahoo! Answers.

**Figure 3:**
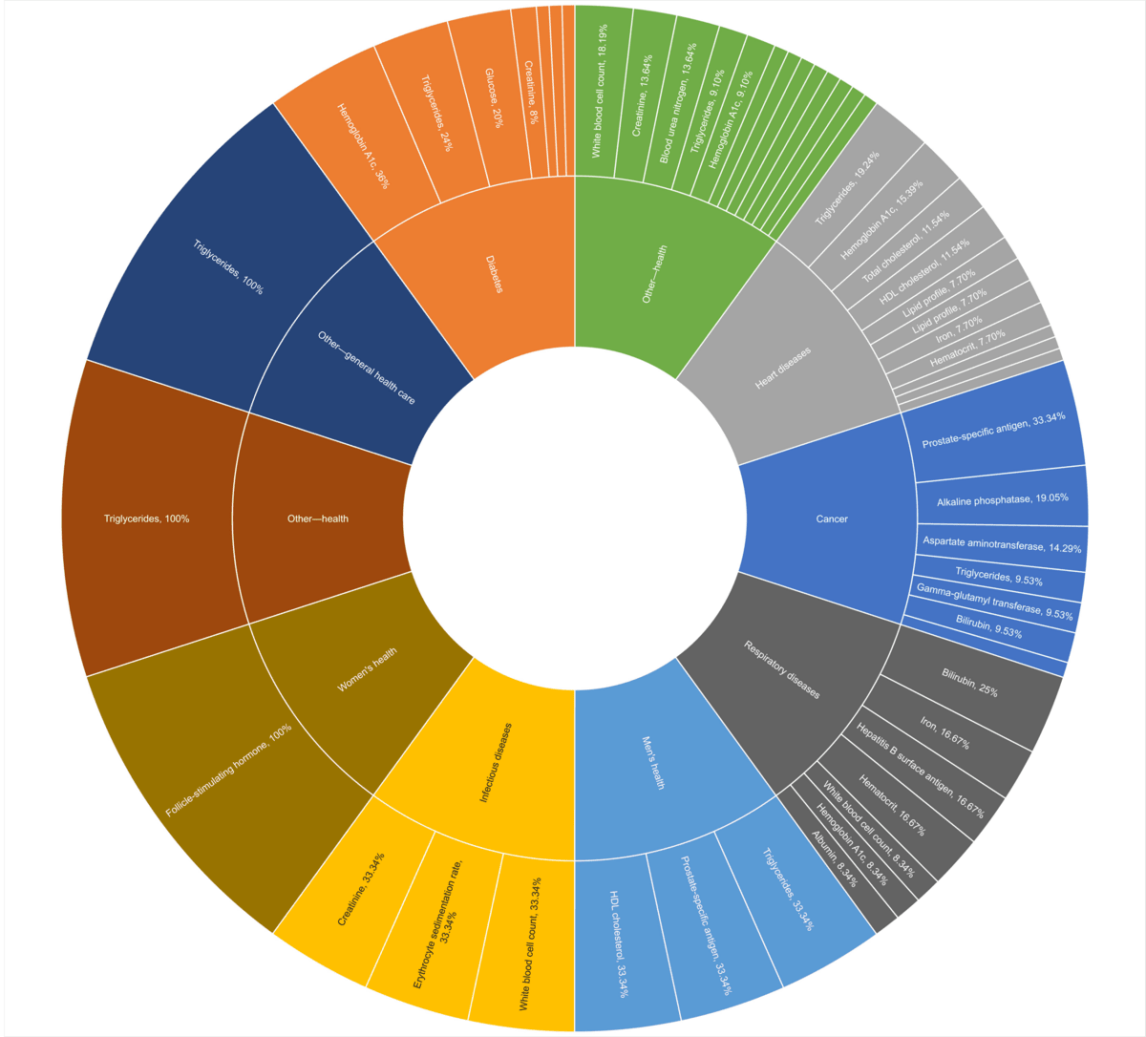
Frequency of 26 laboratory tests for 10 medical conditions in the selected 53 laboratory test questions. HDL: high-density lipoprotein.

Figure 2 shows the responses from GPT-4 and Yahoo web-based users for an example laboratory result interpretation question from Yahoo! Answers. Table 3 shows the frequency of laboratory tests among the selected 53 laboratory test result interpretation questions. Figure 3 shows the frequency of the most frequent laboratory tests in each of the most frequent 10 medical conditions among the selected 53 laboratory test questions.

**Table 3:** Frequency of laboratory tests in the questions (n=53).

Laboratory test	Frequency, n (%)
Triglycerides	18 (34)
Hemoglobin A_1c_	16 (30)
Prostate-specific antigen	9 (17)
White blood cell count	6 (11)
Iron	6 (11)
Glucose	6 (11)
Creatinine	6 (11)
Alkaline phosphatase	6 (11)
Hematocrit	5 (9)
Bilirubin	5 (9)
Lipid profile	4 (8)
HDL^a^ cholesterol	4 (8)
Aspartate aminotransferase	4 (8)
Total cholesterol	3 (6)
Blood urea nitrogen	3 (6)
Hepatitis B surface antigen	2 (4)
γ-glutamyl transferase	2 (4)
Albumin	2 (4)
Vitamin D	1 (2)
Ketones	1 (2)
Glucose tolerance test	1 (2)
Follicle-stimulating hormone	1 (2)
Estimated glomerular filtration rate	1 (2)
Erythrocyte sedimentation rate	1 (2)
Comprehensive metabolic panel	1 (2)
Anion gap	1 (2)

^a^HDL: high-density lipoprotein.

Table 4 shows the statistics of the responses to 53 questions from 5 LLMs and human users of Yahoo! Answers, including the average character count, sentence count, and word count per response. Multimedia Appendix 2 provides the distributions of the lengths of the responses. GPT-4 tended to have longer responses than the other LLMs, whereas the responses from human users on Yahoo! Answers tended to be shorter with respect to all 3 counts. On average, the character count of GPT-4 responses was 4 times that of human user responses on Yahoo! Answers.

**Table 4:** Statistics of laboratory test result interpretation responses in terms of average character count, sentence count, and word count per response.

Model	Character count, mean (SD)	Sentence count, mean (SD)	Word count, mean (SD)
Yahoo! user answer	515 (621)	6 (7)	90 (107)
MedAlpaca	734 (324)	8 (3)	124 (54)
ORCA_mini	942 (292)	9 (3)	156 (47)
LLaMA 2	1308 (326)	12 (3)	212 (54)
GPT-3.5	2246 (345)	19 (4)	340 (51)
GPT-4	2207 (453)	18 (4)	333 (66)

### Automated Comparison of Similarities in LLM Responses

**Figure 4:**
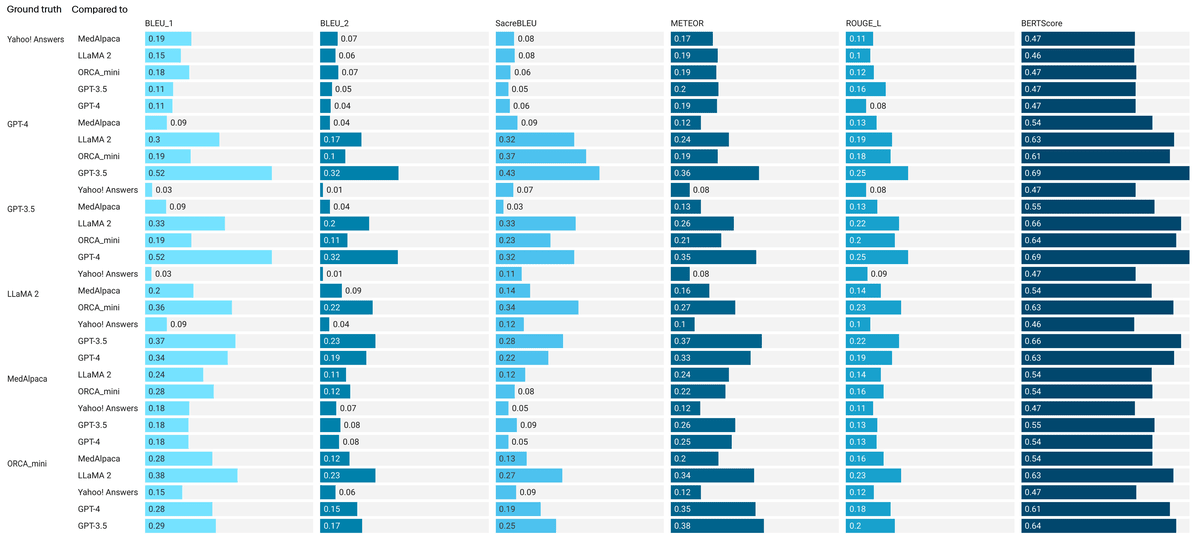
Evaluation results of the responses of the large language models using automatic metrics. BERTScore: Bidirectional Encoder Representations from Transformers Score; BLEU: Bilingual Evaluation Understudy; METEOR: Metric for Evaluation of Translation With Explicit Ordering; ROUGE: Recall-Oriented Understudy for Gisting Evaluation.

Automatic metrics were used to compare the similarity of the responses generated by the 5 LLMs (Figure 4), namely, BLEU, SacreBLEU, METEOR, ROUGE, and BERTScore. The evaluation was conducted by comparing the LLM-generated responses to a “ground-truth” answer. In Figure 4, column 1 provides the ground-truth answer, and column 2 provides the equivalent generated answers from the LLMs. We also included the human answers from Yahoo! Answers for this evaluation. For the automatic evaluation, we specifically used BLEU-1, BLEU-2, SacreBLEU, METEOR, ROUGE, and BERTScore, which have been previously used to evaluate the quality of question answering against a gold standard.

All the metrics ranged from 0.0 to 1.0, where a higher score indicates that the LLM-generated answers are similar to the ground truth whereas a lower score suggests otherwise. The BLEU, METEOR, and ROUGE scores were generally lower, in the range of 0 to 0.37, whereas BERTScore values were generally higher, in the range of 0.46 to 0.63. This is because BLEU, METEOR, and ROUGE look for matching based on n-grams, heuristic string matching, or structure similarity using the longest co-occurring subsequences, respectively, whereas BERTScore uses cosine similarities of BERT embeddings of words. When GPT-4 was the reference answer, the response from GPT-3.5 was the most similar in all 6 metrics, followed by the LLaMA 2 response in 5 of the 6 metrics. Similarly, when GPT-3.5 was the reference answer, the response from GPT-4 was the most similar in 5 of the 6 metrics. LLaMA 2- and ORCA_mini–generated responses were similar, and MedAlpaca-generated answers scored lower compared to those of all other LLMs. Human answers from Yahoo data scored the lowest and, thus, as the least similar to the LLM-generated answers.

Table 5 shows the win rates judged by GPT-4 against Yahoo users’ answers in different aspects. Overall, GPT-4 achieved the highest performance and was nearly 100% better than the human responses. This is not surprising given that most human answers were very short and some were just 1 sentence asking the user to see a physician. GPT-4 and GPT-3.5 were followed by LLaMA 2 and ORCA_mini with 70% to 80% win rates. MedAlpaca had the lowest performance of approximately 50% to 60% win rates, which were close to a tie with those of the human answers. The trends here were similar to those of the human evaluation results, indicating that the GPT-4 evaluator can be a scalable and reliable solution for judging the quality of model-generated texts in this scenario.

**Table 5:** Win rate evaluation results.

Win rate against human answers (evaluated by GPT-4)	Relevance	Correctness	Helpfulness	Less harm
MedAlpaca	50.9	54.9	54.9	54.9
ORCA_mini	78.4	74.5	84.3	84.3
LLaMA 2	82.3	80.3	86.2	70.5
GPT-3.5	98.0	100.0	98.0	96.0
GPT-4	96.0	98.0	98.0	98.0

### Manual Evaluation With Medical Experts

**Figure 5:**
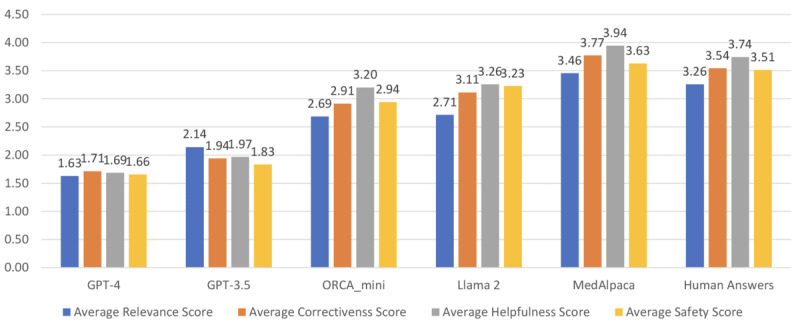
Manual evaluation of the large language model (LLM) and human responses. Lower scores denote better capabilities.

Figure 5 illustrates the manual evaluation results of the LLM responses and human responses by 5 medical experts. Note that a lower value means a higher score. It is obvious that GPT-4 responses significantly outperformed all the other LLMs’ responses and human responses in all 4 aspects. Textbox 2 shows experts’ feedback on the LLM and human responses. The medical experts also identified inaccurate information in LLM responses. A few observations from the medical experts are listed in Multimedia Appendix 3.

**Human experts’ feedback on the large language model and human responses.**

**Large language model or human answer and expert feedback**

- LLaMA 2: “It is a great answer. He was able to explain in details the results. He provides inside on the different differential diagnosis. And provide alternative a management. He shows empathy.”
- LLaMA 2: “Very thorough and thoughtful.”
- ORCA_mini: “It was a great answer. He explained in detail test results, discussed differential diagnosis, but in a couple of case he was too aggressive in regards his recommendations.”
- ORCA_mini: “Standard answers, not the most in depth.”
- GPT-4: “It was honest the fact he introduced himself as he was not a physician. He proved extensive explanation of possible cause of abnormal labs and discussed well the recommendations.”
- GPT-4: “Too wordy at times, gets irrelevant.”
- GPT-3.5: “Strong responses in general.”
- GPT-3.5: “Clear and some way informative and helpful to pts.”
- GPT-3.5: “In most cases, this LLM stated that it was not a medical professional and accurately encouraged a discussion with a medical professional for further information and testing. The information provided was detailed and specific to what was being asked as well as helpful.”
- MedAlpaca: “This statement seems so sure that he felt superficial. It made me feel he did not provide enough information. It felt not safe for the patient.”
- MedAlpaca: “Short and succinct. condescending at times.”
- Human answer: “These were not very helpful or accurate. Most did not state their credentials to know how credible they are. Some of the, if not most, of language learning models gave better answers, though some of the language learning models also claimed to be medical professionals—which isn’t accurate statement either.”
- Human answer: “Usually focused on one aspect of the scenario, not helpful in comprehensive care. focused on isolated lab value, with minimal evidence—these can be harmful responses for patients.”
- Human answer: “These are really bad answers.”
- Human answer: “Some of the answer were helpful, other not much, and other offering options that might not need to be indicated.”

## Discussion

### Principal Findings

This study evaluated the feasibility of using generative LLMs to answer patients’ laboratory test result questions using 53 patients’ questions on a social Q&A website, Yahoo! Answers. On the basis of the results of our study, GPT-4 outperformed other similar LLMs (ie, GPT-3.5, LLaMA 2, ORCA_mini, and MedAlpaca) according to both automated metrics and manual evaluation. In particular, GPT-4 always provided disclaimers, possibly to avoid legal issues. However, GPT-4 responses may also suffer from lack of interpretation of one’s medical context, incorrect statements, and lack of references.

Recent studies [26,27] regarding the use of LLMs to answer laboratory test result questions from medical professionals found that ChatGPT may give superficial or incorrect answers to laboratory test result–related questions and can only provide accurate answers to approximately 50% of questions [26]. They also found that ChatGPT’s responses were not sufficiently tailored to the case or clinical questions to be useful for clinical consultation. For instance, diagnoses of liver injury were made solely based on γ-glutamyl transferase levels without considering other liver enzyme indicators. In addition, high levels of glucose and glycated hemoglobin (HbA_1c_) were both identified as indicative of diabetes regardless of whether HbA_1c_ levels were normal or elevated. These studies also highlighted that GPT-4 failed to account for preanalytical factors such as fasting status for glucose tests and struggled to differentiate between abnormal and critically abnormal laboratory test values. Our study observed similar patterns, where a normal HbA_1c_ level coupled with high glucose levels led to a diabetes prediction and critically low iron levels were merely classified as abnormal.

In addition, our findings also show that GPT-4 accurately distinguished between normal, prediabetic, and diabetic HbA_1c_ ranges considering fasting glucose levels and preanalytical conditions such as fasting status. Furthermore, in cases of elevated bilirubin levels, GPT-4 correctly associated them with potential jaundice citing the patient’s yellow eye discoloration and appropriately considered a comprehensive set of laboratory test results—including elevated liver enzymes and bilirubin levels—and significant alcohol intake history to recommend diagnoses such as alcoholic liver disease, hepatitis, bile duct obstruction, and liver cancer.

On the basis of our observation with the limited number of questions, we found that patients’ questions are often less complex than professionals’ questions, making ChatGPT more likely to provide an adequately accurate answer to such questions. In our manual evaluation of 7 selected patients’ laboratory test result questions, 91% (32/35) of the ratings from 5 medical experts on GPT-4’s response accuracy were either 1 (*very high*) or 2 (*high*).

Through this study, we gained insights into the challenges of using generative LLMs to answer patients’ laboratory test result–related questions and provide suggestions to mitigate these challenges. First, when asking laboratory test result questions on social Q&A websites, patients tend to focus on laboratory test results but may not provide pertinent information needed for result interpretation. In the real-world clinical setting, to fully evaluate the results, clinicians may need to evaluate the medical history of a patient and examine the trends of the laboratory test results over time. This shows that, to allow LLMs to provide a more thorough evaluation of laboratory test results, the question prompts may need to be augmented with additional information. As such, LLMs could be useful in prompting patients to provide additional information. A possible question prompt would be the following: “What additional information or data would you need to provide a more accurate diagnosis for me?”

Second, we found that it is important to understand the limitations of LLMs when answering laboratory test–related questions. As general-purpose generative AI models, they should be used to explain common terminologies and test purposes; clarify the typical reference ranges for common laboratory tests and what it might mean to have values outside these ranges; and offer general interpretation of laboratory test results, such as what it might mean to have high or low levels in certain common laboratory tests. On the basis of our findings, LLMs, especially GPT-4, can provide a basic interpretation of laboratory test results without reference ranges in the question prompts. LLMs could also be used to suggest what questions to ask health care providers. They should not be used for diagnostic purposes or treatment advice. All laboratory test results should be interpreted by a health care professional who can consider the full context of one’s health. For providers, LLMs could also be used as an educational tool for laboratory professionals, providing real-time information and explanations of laboratory techniques. When using LLMs for laboratory test result interpretation, it is important to consider the ethical and practical implications, including data privacy, the need for human oversight, and the potential for AI to both enhance and disrupt clinical workflows.

Third, we found it challenging to evaluate laboratory test result questions using Q&A pairs from social Q&A websites such as Yahoo! Answers. This is mainly because the answers provided by web-based users (who may not be medical professionals) were generally short, often focused on one aspect of the question or isolated laboratory tests, possibly opinionated, and possibly inaccurate with minimal evidence. Therefore, it is unlikely that human answers from social Q&A websites can be used as a gold standard to evaluate LLM answers. We found that GPT-4 can provide comprehensive, thoughtful, sympathetic, and fairly accurate interpretation of individual laboratory tests, but it still suffers from a number of problems: (1) LLM answers are not individualized, (2) it is not clear what are the sources LLMs use to generate the answers, (3) LLMs do not ask clarifying questions if the provided prompts do not contain important information for LLMs to generate responses, and (4) validation by medical experts is needed to reduce hallucination and fill in missing information to ensure the quality of the responses.

### Future Directions

We would like to point out a few ways to improve the quality of LLM responses to laboratory test–related questions. First, the interpretation of certain laboratory tests is dependent on age group, gender, and possibly other conditions pertaining to particular population subgroups (eg, pregnant women), but LLMs do not ask clarifying questions, so it is important to enrich the question prompts with necessary information available in electronic health records or ask patients to provide necessary information for more accurate interpretation. Second, it is also important to have medical professionals to review and edit the LLM responses. For example, we found that LLaMA 2 self-identified as a “health expert,” which is obviously problematic if such responses were directly sent to patients. Therefore, it is important to postprocess the responses to highlight sentences that are risky. Third, LLMs are sensitive to question prompts. We could study different prompt engineering and structuring strategies (eg, role prompting and chain of thought) and evaluate whether these prompting approaches would improve the quality of the answers. Fourth, one could also collect clinical guidelines that provide credible laboratory result interpretation to further train LLMs to improve answer quality. We could then leverage the retrieval-augmented generation approach to allow LLMs to generate responses from a limited set of credible information sources [52]. Fifth, we could evaluate the confidence level of the sentences in the responses. Sixth, a gold-standard benchmark Q&A data set for laboratory result interpretation could be developed to allow the community to advance with different augmentation approaches.

### Limitations

A few limitations should be noted in this study. First, the ChatGPT web version is nondeterministic in that the same prompt may generate different responses when used by different users. Second, the sample size for the human evaluation was small. Nonetheless, this study produced evidence that LLMs such as GPT-4 can be a promising tool for filling the information gap for understanding laboratory tests and various approaches can be used to enhance the quality of the responses.

### Conclusions

In this study, we evaluated the feasibility of using generative LLMs to answer common laboratory test result interpretation questions from patients. We generated responses from 5 LLMs—ChatGPT (GPT-4 version and GPT-3.5 version), LLaMA 2, MedAlpaca, and ORCA_mini—for laboratory test questions selected from Yahoo! Answers and evaluated these responses using both automated metrics and manual evaluation. We found that GPT-4 performed better compared to the other LLMs in generating more accurate, helpful, relevant, and safe answers to these questions. We also identified a number of ways to improve the quality of LLM responses from both the prompt and response sides.

## Appendix

Multimedia Appendix 1 The responses generated by the 5 large language models and the human answers from Yahoo users.

Multimedia Appendix 2 Distribution of the lengths of the responses.

Multimedia Appendix 3 A few observations from the medical experts regarding the accuracy of the large language model responses.

## Abbreviations

AI: artificial intelligence
autoML: automated machine learning
BERTScore: Bidirectional Encoder Representations from Transformers Score
BioBERT: biomedical Bidirectional Encoder Representations from Transformers
BLEU: Bilingual Evaluation Understudy
ClinicalBERT: clinical Bidirectional Encoder Representations from Transformers
HbA_1c_: glycated hemoglobin
ICC: intraclass correlation coefficient
LLM: large language model
METEOR: Metric for Evaluation of Translation With Explicit Ordering
PubMedBERT: PubMed-trained Bidirectional Encoder Representations from Transformers
Q&A: question-and-answer
ROUGE: Recall-Oriented Understudy for Gisting Evaluation
SciBERT: scientific Bidirectional Encoder Representations from Transformers

## Data Availability

The data sets generated during and analyzed during this study are available from the corresponding author on reasonable request.
