# Supplementary material for: Quality of Answers of Generative Large Language Models Versus Peer Users for Interpreting Laboratory Test Results for Lay Patients: Evaluation Study
Source: J Med Internet Res. 2024 Apr 17;26:e56655. doi: 10.2196/56655 (PMC11063893; doi:10.2196/56655)
Supplement: Multimedia Appendix 2 [file jmir_v26i1e56655_app2.pdf]

Multimedia Appendix II

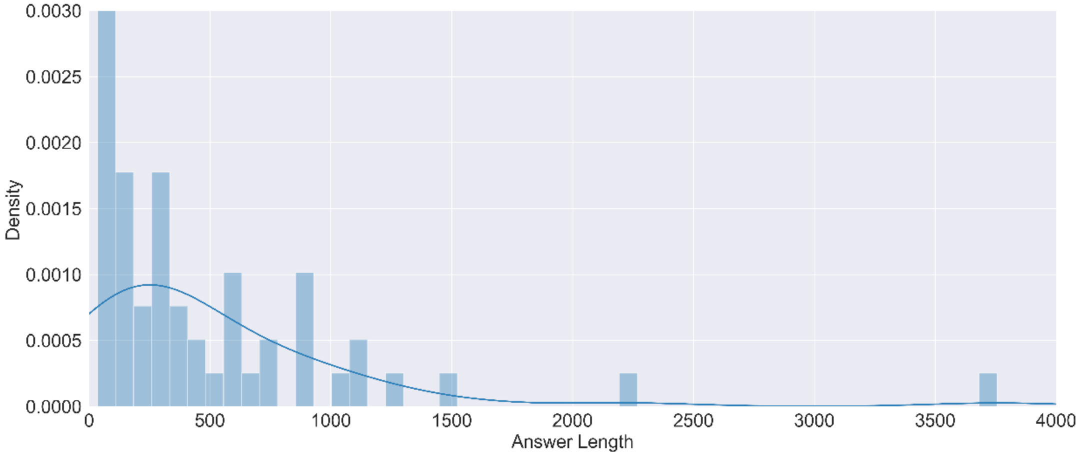

Figure S1. Distribution of lengths of Yahoo user's responses

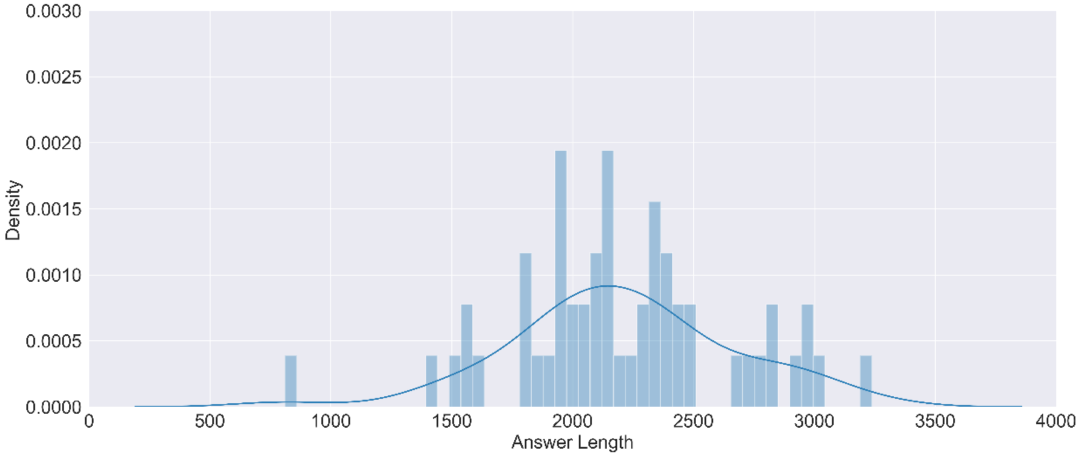

Figure S2. Distribution of lengths of GPT-4's responses

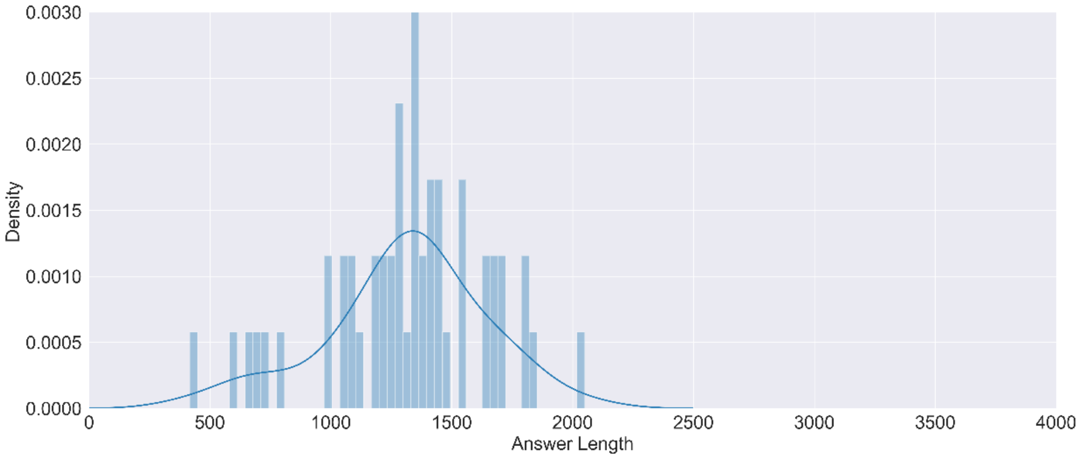

Figure S3. Distribution of lengths of LLaMA 2 responses

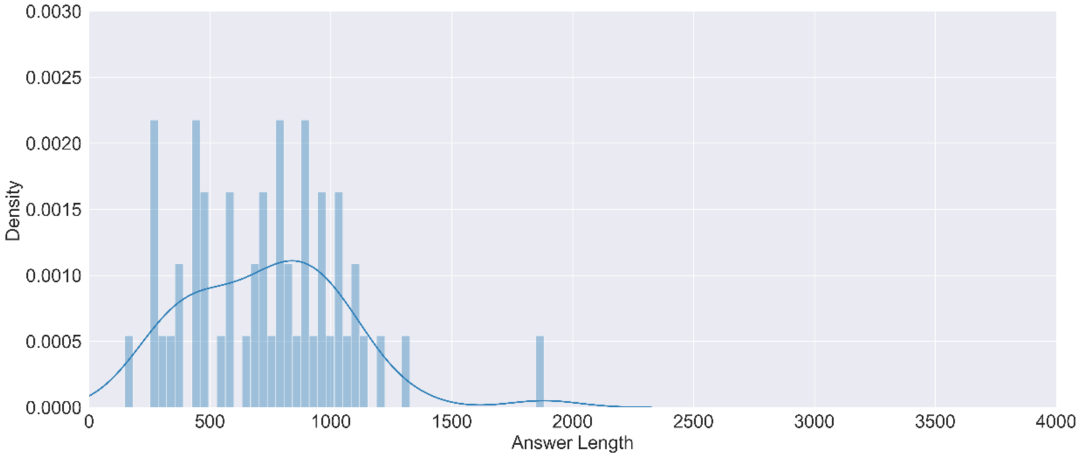

Figure S4. Distribution of lengths of MedAlpaca responses

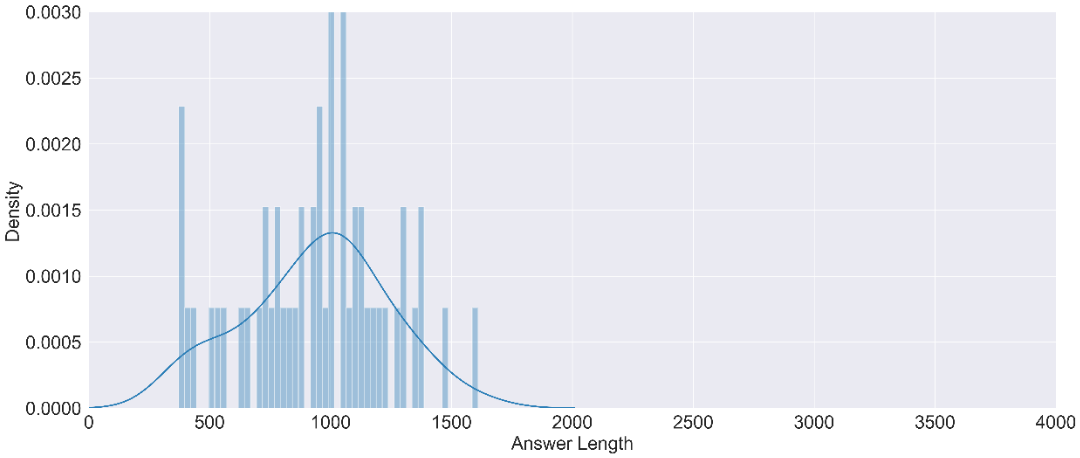

Figure S5. Distribution of lengths of ORCA Mini responses

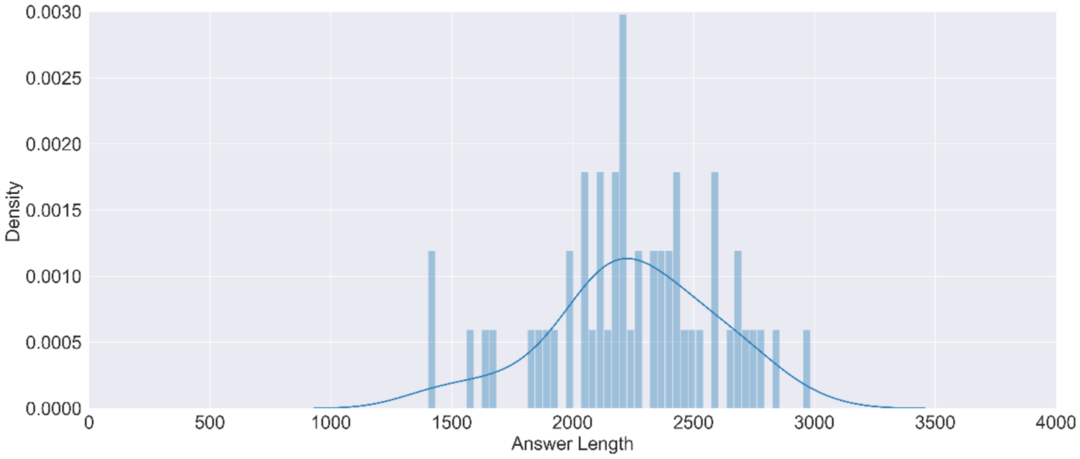

Figure S6. Distribution of lengths of GPT-3.5's responses

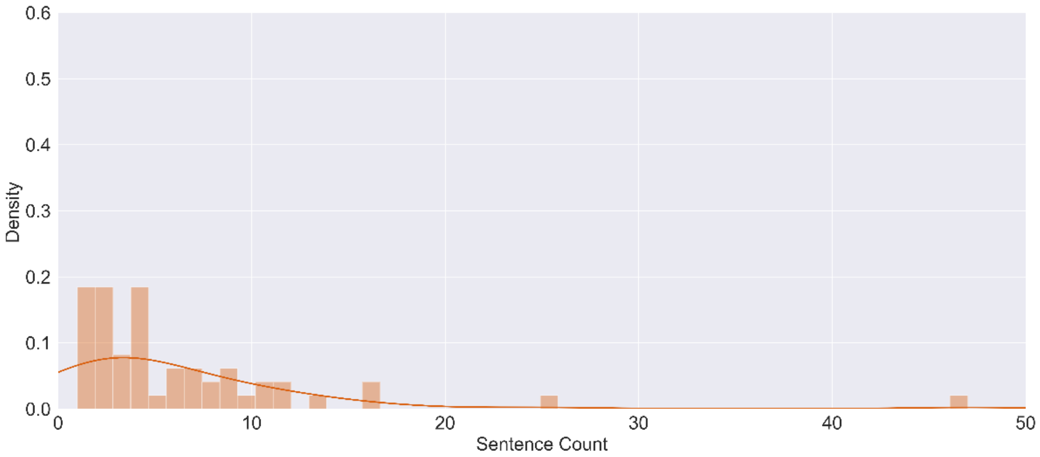

Figure S7. Distribution of sentence count of Yahoo user's responses

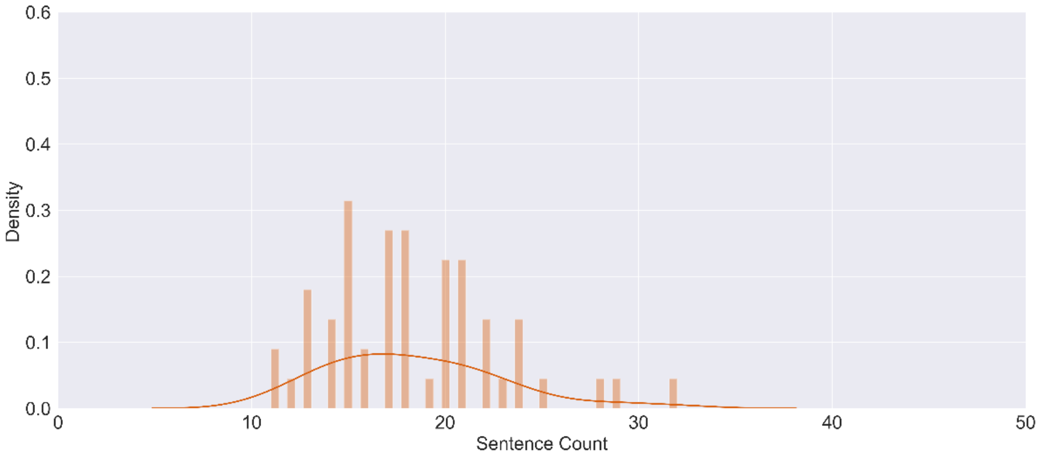

Figure S8. Distribution of sentence count of GPT-4 responses

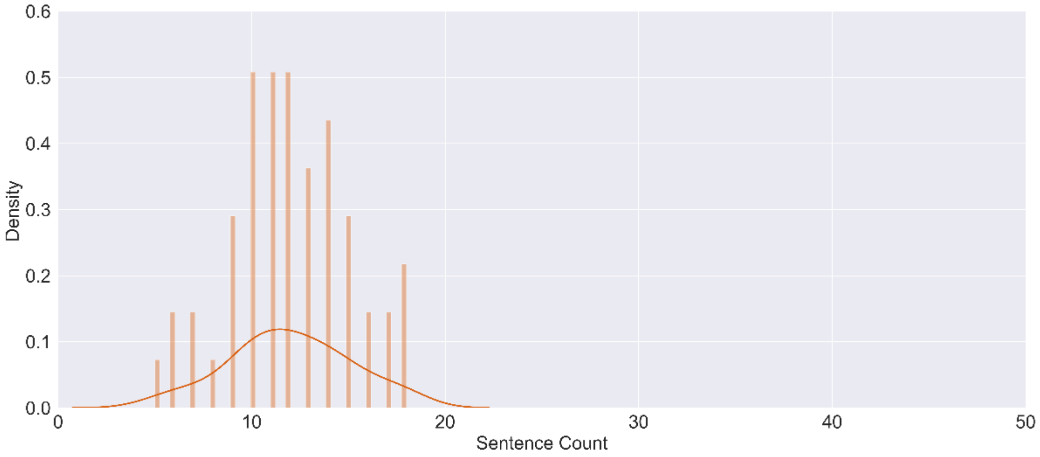

Figure S9. Distribution of sentence count of LLaMA 2 responses

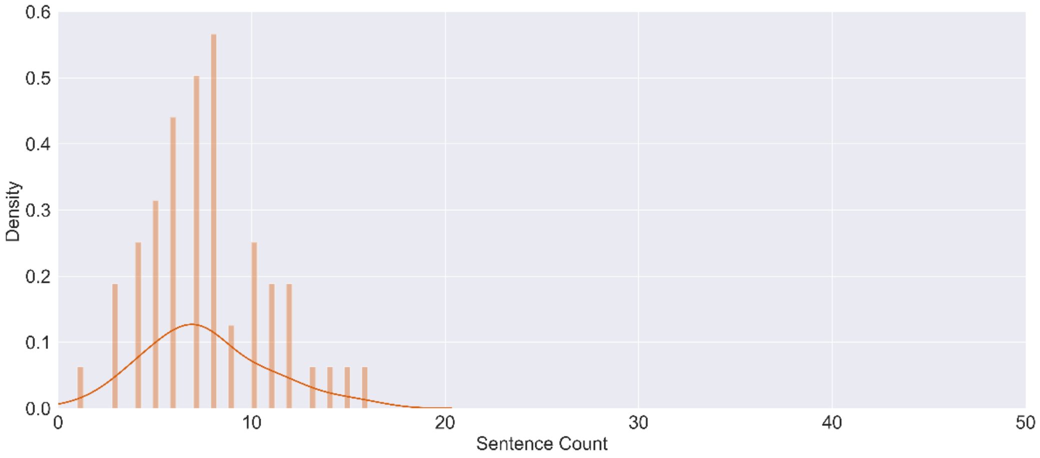

Figure S10. Distribution of sentence count of MedAlpaca responses

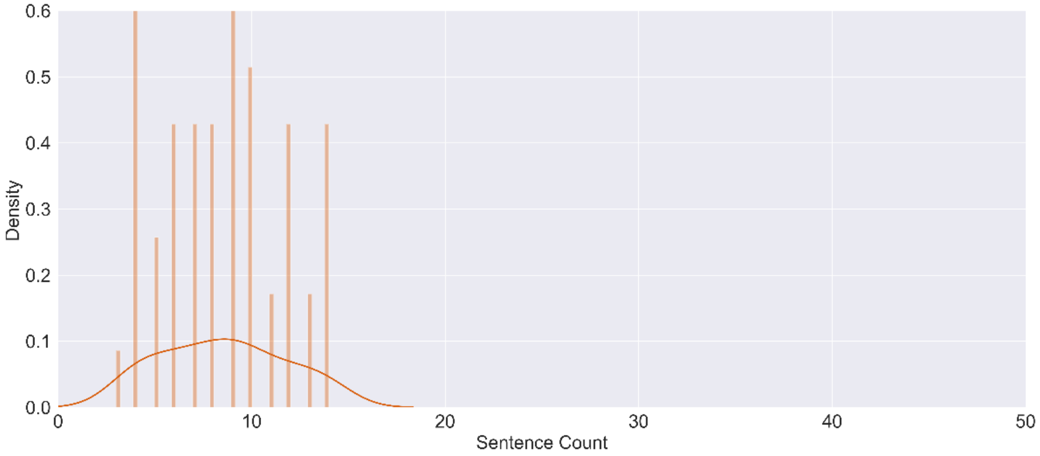

Figure S11. Distribution of sentence count of ORCA Mini responses

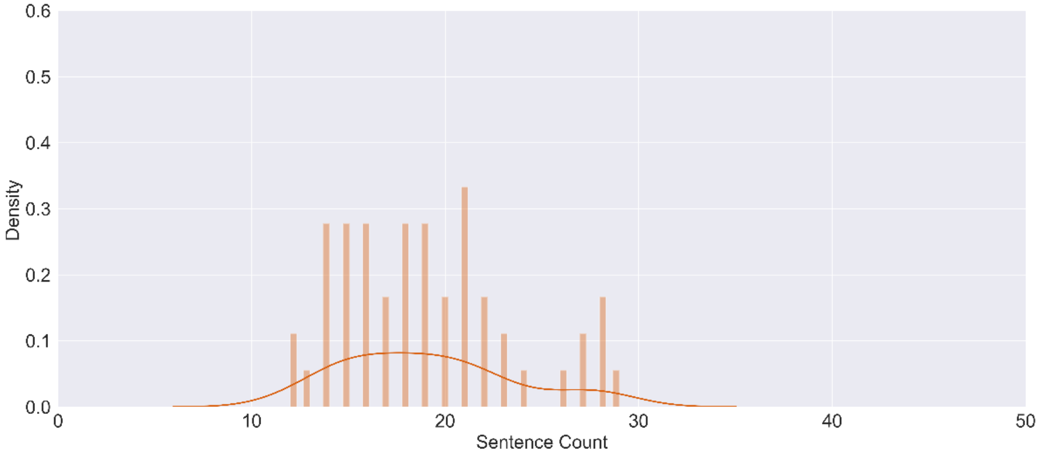

Figure S12. Distribution of sentence count of GPT-3.5 responses

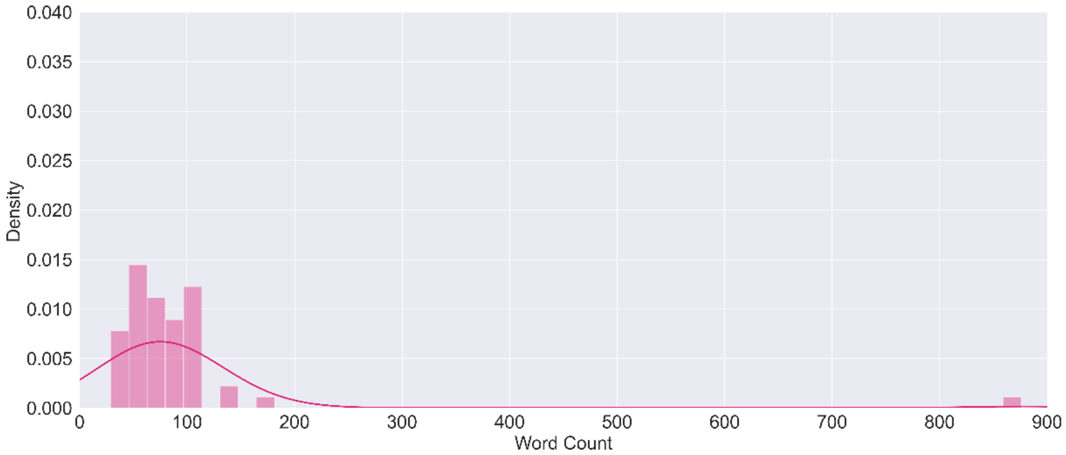

Figure S13. Distribution of word count of Yahoo user's responses

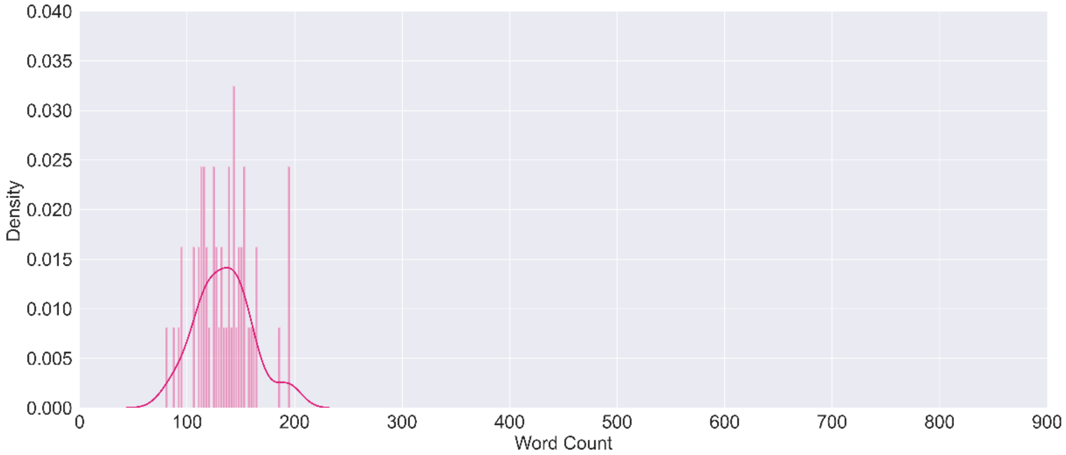

Figure S14. Distribution of word count of GPT-4 responses

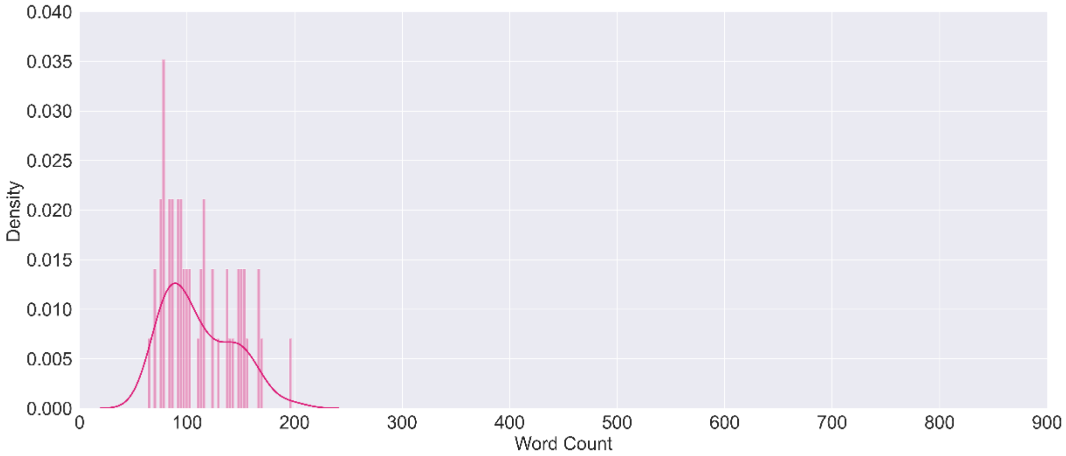

Figure S15. Distribution of word count of LLaMA 2 responses

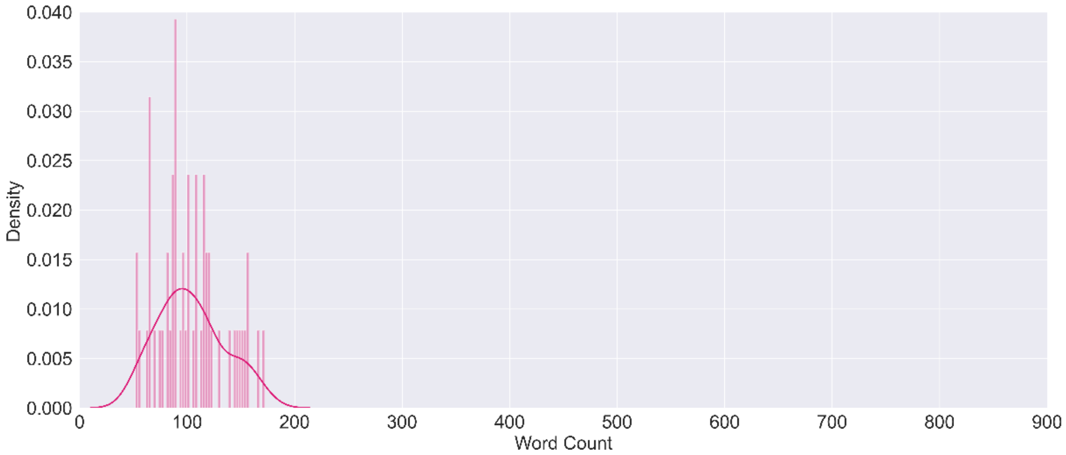

Figure S16. Distribution of wordcount of MedAlpaca responses

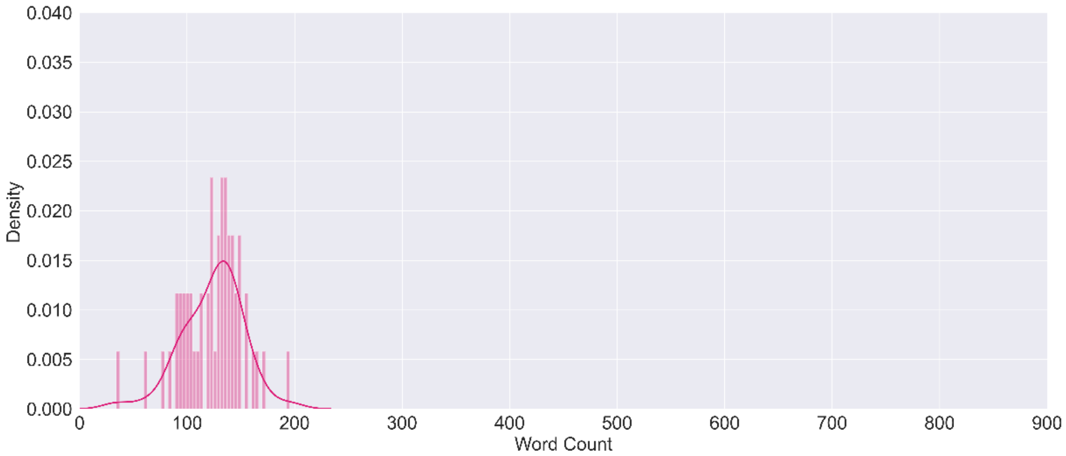

Figure S17. Distribution of word count of ORCA Mini responses

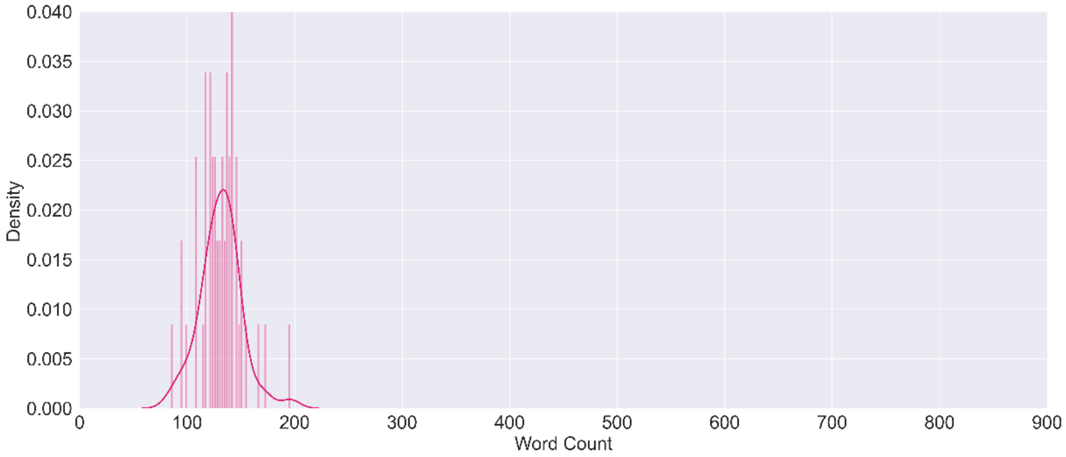

Figure S18. Distribution of word count of GPT 3.5 responses
