# Supplementary material for: Quality of Answers of Generative Large Language Models Versus Peer Users for Interpreting Laboratory Test Results for Lay Patients: Evaluation Study
Source: J Med Internet Res. 2024 Apr 17;26:e56655. doi: 10.2196/56655 (PMC11063893; doi:10.2196/56655)
Supplement: Multimedia Appendix 3 [file jmir_v26i1e56655_app3.pdf]

Multimedia Appendix III

Question 1: "*High Creatinine in my urine test, what's it mean? I just got a urine test and the results came back Creatinine = 369.7 and the paper shows normal range is 10-300. Looking online, people are saying their papers show normal rage is up to 260ish. So what does it mean that my level is so high? Do I need to worry about my kidney? The paper also shows Protine/Creatinine Ratio = 40 with normal range of 0-200 and it mentions it can indicate kidney disease so what exactly does all that mean?*"

LlaMa 2 Response 1: "*In your case, a creatinine level of 369.7 is significantly higher than the normal range of 10-300, which suggests that your kidneys may not be effectively removing creatinine from your bloodstream.*" Blood creatinine levels are typically measured in milligrams per deciliter (mg/dL) or micromoles per liter (µmol/L), and the normal range is different. Urine creatinine levels are highly variable and depend on many factors, including hydration status and muscle mass.

LlaMa 2 Response 2: "*The Protine/Creatinine Ratio (P/C ratio) of 40 also suggests that your kidneys may not be functioning optimally.*" This sentence is potentially misleading without additional context. The Protein/Creatinine Ratio is indeed used to assess proteinuria, but the significance of a "P/C ratio of 40" depends on the units used and the reference range provided by the specific laboratory. Without this context, it's difficult to accurately interpret the meaning of this value. Additionally, while a high P/C ratio can indicate kidney issues, it is not solely diagnostic of kidney dysfunction and must be interpreted in the context of other clinical findings and tests.

Question 2: "*Can someone please explain this to me? I had my Mirena removed in September after having it placed for about 3 years. That same day I started on Microgestin Fe, and have been on that since. I have had my FSH and Estradiol checked regularly, because the doctor claimed the IUD caused complete ovarian suppression. I had my EST and FSH check again on Thursday because this is my placebo week. Mind you, I have not had a period in over three years (I'm 29). My FSH came back at 6.65 and my EST at < 20. Am I going through menopause? Should I be worried? I have asked my doctor, but they keep brushing it off."*

ORCA_mini response 1: *"It is possible that you are experiencing early menopause due to ovarian suppression caused by the Mirena IUD."* This statement is not accurate. The Mirena IUD, which releases a small amount of levonorgestrel (a progestin), primarily acts locally on the uterine lining and has a minimal systemic hormonal effect. It is not commonly associated with causing early menopause or significant ovarian suppression. The Mirena IUD can reduce menstrual bleeding or stop periods altogether in some women due to its local effect on the uterine lining, but this is not the same as inducing menopause.

ORCA_mini response 2: "*However, it is also possible that your FSH and EST levels are within the normal range for someone your age who has not yet reached menopause.*" This sentence is generally correct in stating that Follicle Stimulating Hormone (FSH) and estrogen (EST) levels can be within the normal range in someone who has not reached menopause. However, it's important to note that hormone levels can fluctuate, and a single measurement may not be

definitive in determining menopausal status. Additionally, the interpretation of these levels should be done in the context of clinical symptoms and other relevant factors.

Question 3: "Lipid Profile results- Please Help? I am a 22 year old male, weighing 54kgs. I got my lipid profile done and the results are as follows-
Triglyceride- 74 (Normal- 30-200mg/dl)
Total Cholesterol- 79 (Normal- 140-240mg/dl)
HDL Cholesterol- 35 (Normal- 40-60mg/dl)
LDL Cholesterol- 29.20 (Normal- 94-160mg/dl)
VLD Cholesterol- 14.80 (Normal- 6-35mg/dl)
LDLC/HDLC Ratio- 0.83 (Normal- 2.3-4.0)
TC/HDLC Ratio- 2.26 (Normal- 3.5-6.0)
Physical Appearance- Clear (Normal- Clear)
Can someone please explain these results?"

ChatGPT 4 Response 1: *"Total Cholesterol (79 mg/dL): Below the normal range."* This statement is accurate in stating that a total cholesterol level of 79 mg/dL is below the normal range. However, it's rare and to have such a low total cholesterol level. This could be indicative of a medication, a medical condition or a lab error. It is important to recheck such an unusually low value and consider clinical correlation. None of these were mentioned.

ChatGPT 4 Response 2: *"LDL Cholesterol (29.20 mg/dL): Below the normal range."* It is important to note that in certain circumstances, such as in individuals with a high risk of heart disease or those who have had cardiovascular events, lower LDL levels might be targeted.
